# Structure and Frictional Properties of Ultrahard AlMgB_14_ Thin Coatings

**DOI:** 10.3390/nano13101589

**Published:** 2023-05-09

**Authors:** Dmitrii Tkachev, Ilya Zhukov, Pavel Nikitin, Victor Sachkov, Alexander Vorozhtsov

**Affiliations:** Laboratory of Metallurgy Nanotechnologies, National Research Tomsk State University, Lenin Avenue 36, 634050 Tomsk, Russia; d.tkachev11@gmail.com (D.T.); upavelru@yandex.ru (P.N.); vicsachkov@gmail.com (V.S.); abv1953@mail.ru (A.V.)

**Keywords:** ceramic coatings, friction, hardness, physical vapor deposition, high frequency plasma sputtering

## Abstract

This paper presents the results of studies on AlMgB_14_-based ceramic coatings deposited on WC-Co hard alloy substrates using RF plasma sputtering. The aim of this work is to study the structure, phase composition, and mechanical properties of AlMgB_14_-based coatings depending on the sputtering mode. According to the results of the microstructural study, the bias voltage applied to the substrate during the sputtering process significantly contributed to the formation of the coating morphology. Based on the results of compositional and structural studies by energy dispersive X-ray spectroscopy, X-ray diffraction, and Raman spectroscopy, it was found that the coatings are composed of nanocrystalline B_12_ icosahedrons distributed in an amorphous matrix consisting of Al, Mg, B, and O elements. The nanohardness of the coatings varied from 24 GPa to 37 GPa. The maximum value of the hardness together with the lowest coefficient of friction (COF) equal to 0.12 and wear resistance of 7.5 × 10^−5^ mm^3^/N·m were obtained for the coating sputtered at a bias voltage of 100 V. Compared with the COF of the original hard alloy substrate, which is equal to 0.31, it can be concluded that the AlMgB_14_-based coatings could reduce the COF of WC-based hard alloys by more than two times. The hardness and tribological properties of the coatings obtained in this study are in good agreement with the properties of AlMgB_14_-based materials obtained by other methods reported in the literature.

## 1. Introduction

Improving wear resistance while reducing the coefficient of friction of the surface of materials used in the manufacture of friction pairs and machining tools is a current scientific and technical challenge. The most widespread and promising method is to modify the surface of parts by sputtering coatings, which can significantly increase hardness and wear resistance and reduce the coefficient of friction compared to the base material. Currently, a high level of development has been achieved in this direction. Nitride-based coatings such as Si_3_N_4_, TiN, and c-BN [1,2]; carbides: SiC and WC [1,3]; oxides: Al_2_O_3_ and Cr_2_O_3_ [4]; borides: TiB_2_ [5]; and diamond-like carbon (DLC) coatings [6] are widely used. Research into fundamentally new materials that offer a multiple reduction in the coefficient of friction, while simultaneously increasing hardness and wear resistance, is of great scientific importance. Among these materials, aluminum magnesium boride AlMgB_14_-based ceramics could be highlighted [7,8,9].

According to [8], the coefficient of friction of AlMgB_14_-based coatings can reach 0.02 with lubrication, while the hardness reaches 35 GPa and higher. According to [9], AlMgB_14_-based coatings have a lower coefficient of friction than the “industry leading” DLC coating. In addition to testing the coefficient of friction under laboratory conditions, the authors [9] demonstrated the effectiveness of AlMgB_14_-based coatings when applied to the friction pairs of a hydraulic pump. The coefficient of friction of AlMgB_14_-based coatings is reduced both by their high hardness and by the realization of the so-called self-lubricating effect. This effect consists in the formation of a thin layer of B(OH)_3_ on the surface of AlMgB_14_ in a humid atmosphere. This layer consists of B(OH)_3_ atomic layers connected together by weak van der Waals bonds. Thus, it provides a reduction in the resistance to the applied frictional force and reduces the coefficient of friction [9]. In addition, AlMgB_14_ is resistant to chemically aggressive environments and high temperatures and has a coefficient of thermal expansion close to that of steel and hard alloys, ensuring minimal stress between the coating and the substrate.

AlMgB_14_-based materials are prepared by sintering, self-propagating high-temperature synthesis (SHS), hot pressing, and spark plasma sintering [10,11,12]. AlMgB_14_-based coatings can be obtained using physical vapor deposition [13,14,15,16]. Both targets consist of pre-synthesized powder materials [13] and stoichiometric elemental powder compositions [14], or separate elemental targets [15,16] can be used to obtain AlMgB_14_-based coatings. For example, in [15], the DC magnetron sputtering method was used to obtain coatings using three Al-, Mg-, and B-based targets. Sputtering of the AlMgB_14_-based coating using separate elemental targets is a complicated technological process, as it requires fine experimental tuning of deposition technological modes to control the ratio of the sputtered elements on the substrate surface. In [16], to simplify the technological process, a target based on the intermetallic AlMg alloy was used instead of separate Al- and Mg-based targets. However, regardless of the target type and sputtering mode, all of the coatings reported in [13,14,15,16] were formed in the X-ray amorphous state.

Thus, despite the high hardness, wear resistance, and low coefficient of friction, the sputtering processes, mechanisms of structure formation, and physical and mechanical properties of AlMgB_14_ coatings are still unclear. In addition, the scientific knowledge of AlMgB_14_-based coatings is not sufficient for their transition to industrial application. This determines the relevance of scientific studies aimed at the development of initial production methods for AlMgB_14_-based materials and the study of the structure, phase composition, and mechanical and tribological properties of AlMgB_14_-based coatings. Thus, the purpose of this study is to investigate the microstructure, crystal structure, hardness, coefficient of friction, and wear resistance of AlMgB_14_-based coatings obtained by high-frequency plasma sputtering of a previously synthesized via self-propagating high-temperature synthesis (SHS) AlMgB_14_-based powder target.

## 2. Materials and Methods

### 2.1. Process for Obtaining AlMgB_14_-Based Ceramic Powders

The starting AlMgB_14_-based powder material was obtained by the SHS method developed in our previous work [17] from a premix of Al_12_Mg_17_ alloy and amorphous boron powders in the stoichiometric atomic ratio of 2:14. The SHS was performed using a Ti-Si chemical furnace as the heat energy source. First, the stoichiometric Al_12_Mg_17_:B premix was mechanically activated for 3 hours in an argon atmosphere with steel grinding bodies at a ratio of 3:1 to the mass of the powder to ensure uniform mixing of the components and enhance their reactivity. The powder was then cold-pressed into 23 mm diameter specimens weighing 15 g each. To form a chemical furnace, the donor mixture of titanium and silicon in the stoichiometric ratio for Ti_5_Si_3_ synthesis (74 wt. % Ti + 26 wt. % Si) was poured into a cylindrical cellulose paper container, into which the acceptor sample was placed. The optimum thickness of the chemical furnace was 3 mm, as determined in [17]. The resulting system was placed in a constant pressure reactor. The reactor was evacuated, and then its working space was filled with argon to a pressure of 2 bar. The synthesis reaction was initiated by short-term heating of the upper surface of the system with a molybdenum spiral. After synthesis, the specimen was separated from the chemical furnace and ground to powder by hand in a mortar. The obtained powder was further ground in a planetary ball mill at 14 Hz for 40 min with tungsten carbide grinding bodies at a ratio of 3:1 to the mass of the powder. The ground powder was then sieved through a 40 µm mesh. After grinding and sieving, the obtained AlMgB_14_-based powder had a uniform structure, represented by angular grains of AlMgB_14_. The average particle size of the powder was 3.5 μm. According to the results of X-ray phase analysis, the obtained powder material contained 91 wt. % of AlMgB_14_ phase and 9 wt. % of the spinel MgAl_2_O_4_ phase, formed due to the presence of oxygen in the initial boron powder [17].

### 2.2. RF Plasma Sputtering

**Figure 1:**
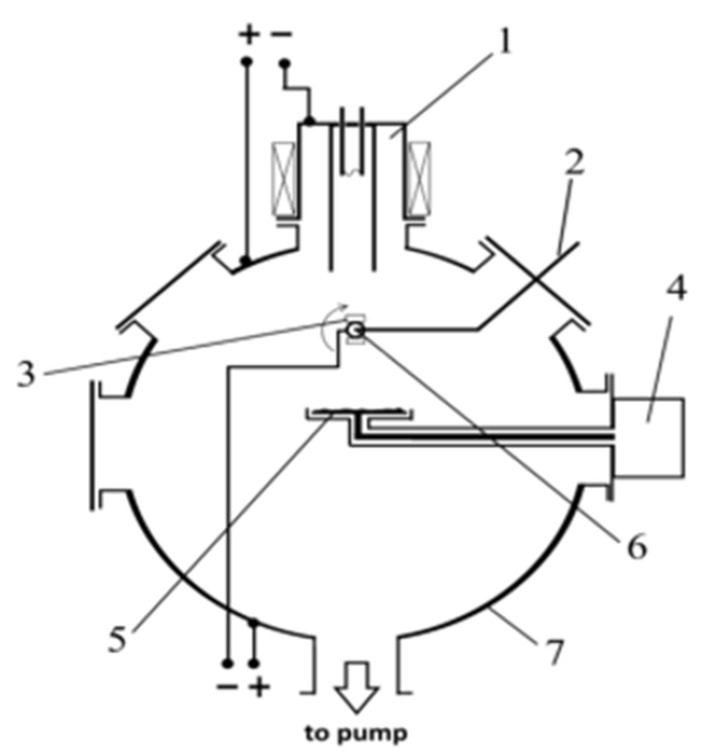
Scheme of the sputtering equipment: 1—Plasma generator; 2—Thermocouple; 3—Sputtering substrate; 4—RF generator; 5—Target; 6—Substrate holder; 7—Vacuum chamber.

For sputtering of AlMgB_14_-based coatings, electron-ion-plasma equipment “COMPLEX” (IHCE SB RAS, Tomsk, Russia) was used [18]. The equipment combines ion-plasma etching of the sample surface and the deposition of coatings from powder or solid targets. A schematic of the sputtering equipment is shown in Figure 1.

The sputtering process consisted of the following steps. The substrate and AlMgB_14_ powder target were placed in a vacuum chamber and evacuated to a pressure of 5 × 10^−3^ Pa. After argon was introduced into the chamber at a pressure of 0.1–0.4 Pa, the plasma generator was turned on. First, a negative bias voltage was applied to the substrate to clean and activate the surface using argon plasma. After the surface was treated and the substrate was heated to 450 °C, the RF generator connected to the target was turned on to initiate the sputtering of the coating material onto the substrate surface. After sputtering, the substrate was cooled to a temperature of less than 100 °C under vacuum. The coatings were sputtered at bias voltages ranging from 35 V to 150 V. Table 1 lists the values of the main sputtering parameters.

### 2.3. Characterization

The microstructures of the coatings were studied using an optical microscope (METAM LV-34, Saint Petersburg, Russia) and a scanning electron microscope (TESCAN Mira, Brno, Czech Republic). The morphology of the coatings was studied using an atomic force microscope (AFM) NT MDT (Moscow, Russia). The elemental composition of the coating cross-section was studied using energy dispersive X-ray spectroscopy (EDX) (Oxford Instrument, Abingdon, UK) during microstructural studies. The crystal structure was studied by X-ray diffraction analysis using a Shimadzu XRD 6000 diffractometer (Kyoto, Japan) with CuKα radiation and Raman spectroscopy using a Renishaw spectrometer (Wotton-under-Edge, UK) at a wavelength of 785 nm. XRD analysis of the coatings was performed in sliding beam mode with a sliding angle of 2°. Nanoindentation of the coatings was performed using a CSM-Instruments (Peseuxm Switzerland) desktop nanoindentation system with a load of 15 mN and exposure time of 5 s. Nanohardness was determined using the Oliver and Farr method [19]. The coefficient of friction and wear rate were determined using the pin-on-disc method with a 100Cr6 steel ball under dry conditions at room temperature with a load of 1 N and test speed of 25 mm/s using a TRIBOtechnic (Clichy, France) tribometer.

## 3. Results

### 3.1. Microstructure

**Figure 2:**
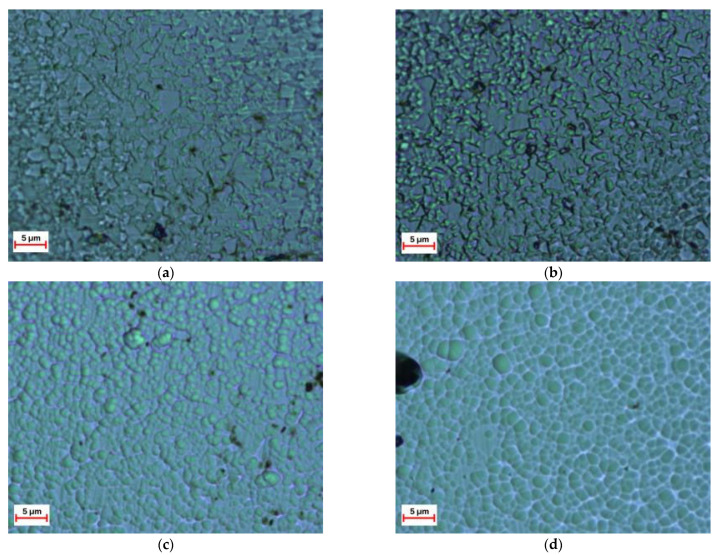
Optical microscopy images of the surface of the AlMgB_14_-based coatings sputtered at different bias voltage: (**a**) 35 V; (**b**) 50 V; (**c**) 100 V; (**d**) 150 V.

Figure 2 shows the surface microstructure images of AlMgB_14_-based coatings obtained at different bias voltages.

As shown in Figure 2, the microstructure of the AlMgB_14_-based coating sputtered at a bias voltage of up to 50 V is represented by angular regions with sizes of 2–5 μm. As the bias voltage increases, the microstructure of the coating surface is represented by rounded clusters. The higher the bias voltage is, the larger are the individual clusters. Thus, for the coating sputtered at a bias voltage of 100 V, the average size of the clusters representing the surface microstructure is 2–3 μm, whereas for the coating sputtered at a bias voltage of 150 V, the size of individual clusters reaches 5 μm.

**Figure 3:**
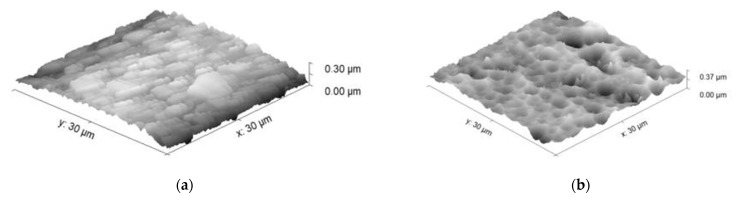
AFM images of the surface microstructure of the AlMgB_14_-based coatings sputtered at different bias voltage: (**a**) 35 V; (**b**) 150 V.

AFM images representing the morphology of the sputtered AlMgB_14_-based coatings are shown in Figure 3.

According to the AFM images (Figure 3), the angular regions formed on the surface of the AlMgB_14_-based coatings sputtered at a bias voltage of 35 V were up to 300 nm in height. As the bias voltage increased up to 150 V, the morphology of the coating surface was represented by spherical cavities with depths of up to 300 nm. The average roughness calculated from the AFM results for the 100 × 100 μm regions is 22.05 nm for the AlMgB_14_-based coating sputtered at a bias voltage of 35 V and 75.3 nm for the coating sputtered at a bias voltage of 150 V.

### 3.2. Composition and Crystal Structure

**Figure 4:**
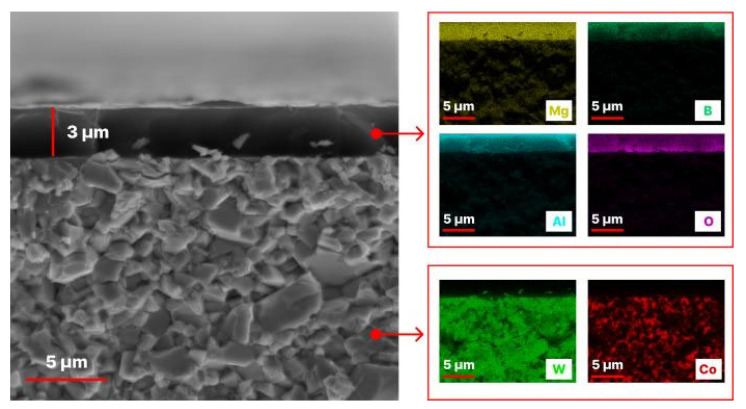
SEM image and EDX mapping of the cross-section of the AlMgB_14_-based coating sputtered at 100 V bias voltage.

Figure 4 shows SEM images and EDX mapping of the cross-section of the AlMgB_14_-based coating obtained at a bias voltage of 100 V.

The SEM image (Figure 4) shows a clear boundary between the coating and substrate. As shown in Figure 4, the sputtered coating was 3 μm thick. The EDX mapping shows that the coating is represented by uniformly distributed elements of the Al-Mg-B system. In addition to Al-Mg-B, oxygen was uniformly distributed in the coating structure. This can be explained by the presence of oxygen in the initial AlMgB_14_ target powder. The EDX maps obtained for W and Co are typical for WC-Co hard alloy substrate materials.

**Figure 5:**
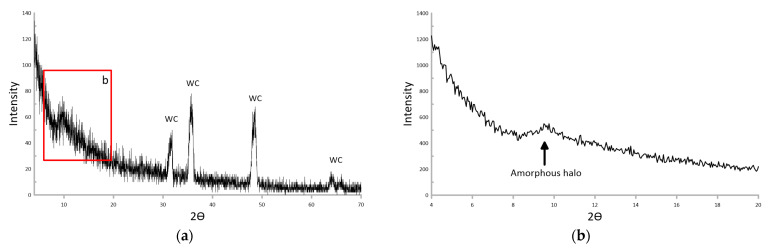
XRD patterns of the AlMgB_14_-based coating sputtered at 100 V bias voltage obtained with different exposure times and angles: (**a**) 1 s for angles 4–70°; (**b**) 10 s for angles 4–20°.

The results of the XRD analysis of the AlMgB_14_-based coating sputtered at a bias voltage of 100 V are shown in Figure 5. The XRD analysis was carried out with a standard exposure time of 1 s (Figure 5a) and with an exposure time of 10 s (Figure 5b) for the angles 4–20° (area marked with the letter “b” in Figure 5a).

According to Figure 5a, the XRD pattern obtained with 1 s exposure contains, in addition to reflexes related to the WC-Co substrate, a broad reflex at low angles in the region of 10°. Based on the XRD pattern shown in Figure 5a, this reflex could refer to both the crystalline and amorphous states. The XRD pattern obtained with a longer exposure of 10 s (Figure 5b) determines the nature of this broad reflex as an amorphous halo. Because the XRD pattern of the AlMgB_14_-based coating sputtered at a bias voltage of 100 V contains only the WC-Co substrate reflexes and an amorphous halo, it can be concluded that the coating was formed in the amorphous state during RF plasma sputtering.

**Figure 6:**
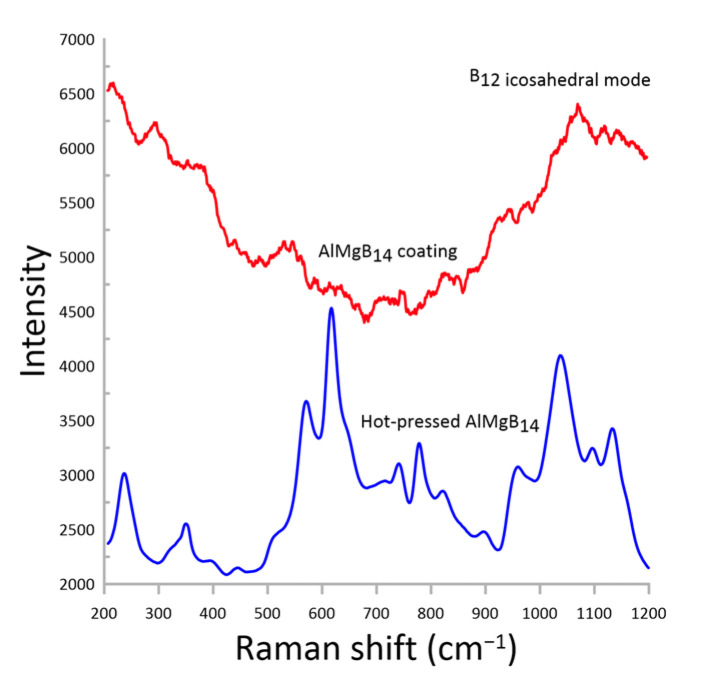
Raman spectra for AlMgB_14_-based coating sputtered at 100 V bias voltage and hot-pressed AlMgB_14_ sample.

Figure 6 shows the Raman spectra of the AlMgB_14_-based coating sputtered at a bias voltage of 100 V. For comparison, the Raman spectra for the hot-pressed AlMgB_14_-based sample (Figure 6, blue line) obtained in our previous work [20] are also shown.

According to Figure 6, the obtained Raman spectra for the AlMgB_14_-based coating did not contain any narrow lines because the coating was formed in the amorphous state. However, there is a broad band from 1000 cm^−1^ to 1200 cm^−1^ in the Raman spectra of both the coating and hot-pressed AlMgB_14_-based materials. According to the literature [21,22,23] this band can be attributed to the icosahedral B-B vibrational modes. This indicated that individual nanocrystalline B_12_ icosahedrons were formed in the amorphous structure of the RF plasma-sputtered AlMgB_14_-based coating.

### 3.3. Mechanical and Tribological Properties

**Figure 7:**
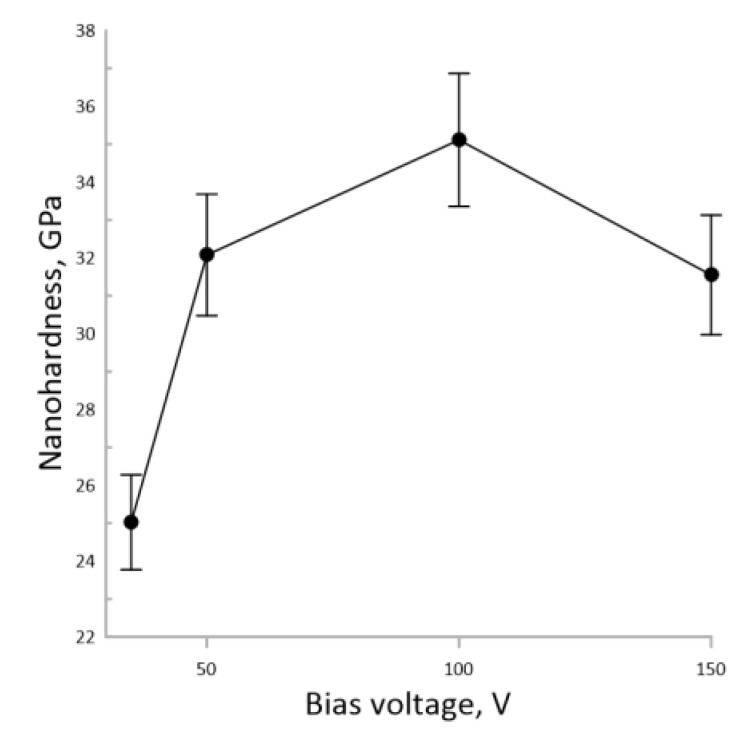
The dependence of the nanohardness of the AlMgB_14_-based coatings on the bias voltage during sputtering.

Figure 7 shows the dependence of the nanohardness of the coatings on RF sputtering bias voltage.

**Figure 8:**
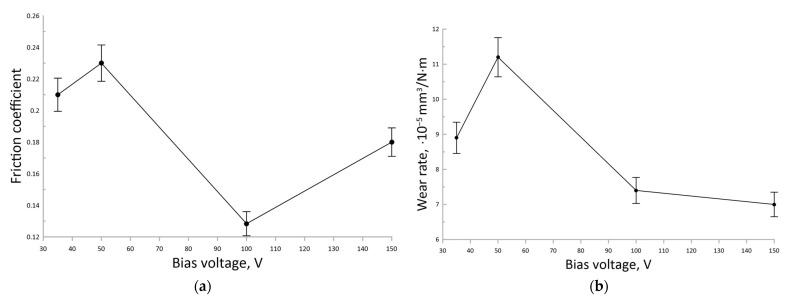
The dependences of the tribological properties of the AlMgB_14_-based coatings on the bias voltage during sputtering: (**a**) Coefficient of friction; (**b**) Wear rate.

According to Figure 7, an increase in the RF sputtering bias voltage up to 100 V leads to an increase in the coating nanohardness up to 37 GPa. With a further increase in the RF sputtering bias voltage to 150 V, the nanohardness decreased to 32 GPa. The dependence of the tribological characteristics of the sputtered coatings is shown in Figure 8.

**Figure 9:**
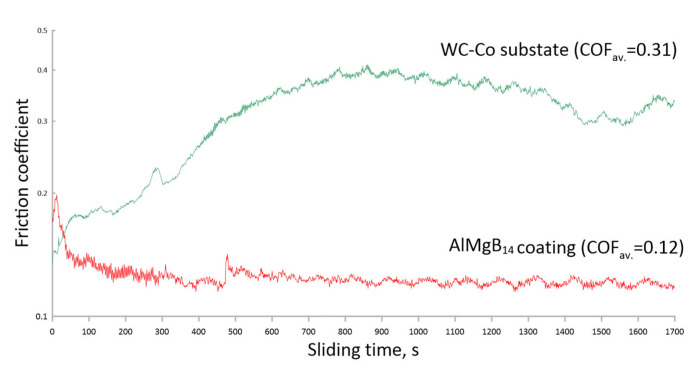
Tribological behaviors of the WC-based substrate and AlMgB_14_-based coating sputtered at 100 V bias voltage.

According to Figure 8a, the COF of the obtained AlMgB_14_-based coatings varies from 0.12 to 0.24 depending on the sputtering bias voltage. When the bias voltage is increased from 35 V to 50 V, the COF increases slightly by a value close to the measurement error. When the bias voltage is subsequently increased to 100 V, the COF decreases significantly to a minimum value of 0.12. For the bias voltage of 150 V, the COF increases again to the average value of 0.18. Thus, except for the maximum nanohardness of 37 GPa, the AlMgB_14_-based coating sputtered at a bias voltage of 100 V has the lowest coefficient of friction equal to 0.12 and a relatively low wear rate of 7.5 × 10^−5^ mm^3^/N·m compared with the coatings sputtered at 35 V, 50 V, and 150 V bias voltages. Figure 9 shows the coefficient of friction as a function of sliding time for the WC-based substrate and the AlMgB_14_-based coating sputtered at 100 V bias voltage.

According to Figure 9, the COF of the WC-Co substrate starts from 0.15 and increases with the time of the tribological test up to an average value of 0.31. The increase in COF is related to the abrasive destruction of the WC-Co surface. The COF of the AlMgB_14_-based coating starts from the higher value of 0.18 and increases sharply to 0.2 with the start of the tribological test, which can be defined as the lapping stage caused by the irregularities of the surface microstructure. After the first 50 s, the COF of the AlMgB_14_-based coating decreased to an average value of 0.12.

## 4. Discussion

Regardless of the RF plasma sputtering mode, according to the results of XRD analysis, the AlMgB_14_-based coatings were formed in the amorphous state. Such an amorphous structure of sputtered coatings is typical for RF plasma sputtering. This can be explained by the highly intensive surface treatment by the ions of the sputtered material. Consequently, a regular crystal structure was not formed. This is in good agreement with the results presented by other authors [15,21,24], which also showed the formation of an amorphous structure of AlMgB_14_-based coatings, regardless of the deposition mode. However, the Raman spectra show the formation of B_12_ icosahedral structures in the amorphous Al-Mg-B matrix, which in turn defines the obtained high hardness values of up to 37 GPa.

The morphology of the coatings was found to be highly dependent on the sputtering mode. For example, at low bias voltages (35 V), the coating surface was represented by the angular regions of the sputtered material. As the bias voltage increased, spherical cavities were formed on the surface of the coatings as the surface treatment by the ions of the sputtered material intensified. As a result, as the RF sputtering bias voltage increases from 35 V to 150 V, the average roughness of the coating increases from 22.05 nm to 75.3 nm.

According to the results of tribological studies, the COF depends non-linearly on the bias voltage. Two factors, surface morphology and hardness, can cause COF changes. Thus, it appears that as the bias voltage increases from 35 V to 50 V, the roughness of the coating increases, which also causes the COF to increase but by an insignificant value, comparable to the measurement error. As the bias voltage is further increased to 100 V, the COF decreases sharply to the lowest value of 0.12. The low COF of the AlMgB_14_-based coating could be explained by its high hardness. Because the hardness of the AlMgB_14_-based coating obtained at a bias voltage of 100 V is higher than that of the coatings obtained at other bias voltages and a substrate, it exhibits less abrasive wear during friction. As a result, the COF of the coating was lower and more stable during the test compared to the substrate (Figure 9).The self-lubricating effect of AlMgB_14_ described in other papers [8,24,25,26,27] could also contribute to the reduction in the coating COF. Therefore, the AlMgB_14_-based coating RF sputtered at a bias voltage of 100 V reduced the COF of the WC-Co substrate by more than a factor of 2.5. Meanwhile, as the bias voltage is subsequently increased, the COF increases again due to a decrease in hardness and an increase in the roughness of the coating.

The properties of the AlMgB_14_-based coatings obtained in this study, compared with those of similar materials reported in the literature, are shown in Table 2.

According to Table 2, the RF-sputtered AlMgB_14_-based coatings have a higher maximum hardness than the AlMgB_14_-based materials studied by other authors. At the same time, the tribological properties of the coatings sputtered in the current study are in good agreement with the tribological properties investigated in other studies. According to other studies, the lowest COF values of AlMgB_14_-based coatings were obtained when a lubricating medium was applied. The wear resistance of the coatings was mainly influenced by the counter ball material and the composition of the target material for coating sputtering. For example, according to [25], the wear rate of the AlMgB_14_-30 wt. % Si increases by one order when tested with a Si_3_N_4_-based ball compared to the wear rate when tested with a steel ball. It is evident that this change in wear rate is caused by an increase in the hardness of the counterbody material. In [8], it was shown that a significant increase in the wear resistance of AlMgB_14_-based coatings and materials was achieved by introducing TiB_2_. It has been reported that the wear rate of AlMgB_14_-50 wt. % TiB_2_ coating is 6.5 × 10^−7^ mm^3^/N·m [8], which is 2 orders lower than the wear rate of AlMgB_14_-based coating without additives obtained in the present work.

## 5. Conclusions

In this work, 3 μm-thick AlMgB_14_-based coatings were deposited on WC-Co hard alloy substrates by RF plasma sputtering with varying bias voltages applied to the substrate. According to the XRD studies, sputtered coatings were formed in an amorphous state. However, the Raman spectra showed the formation of nanocrystalline B_12_ icosahedrons in the amorphous Al-Mg-B coating structure. The morphology of the coating surface and the mechanical and tribological properties depend on the bias voltage applied to the substrate during sputtering. Therefore, as the bias voltage increases from 35 V to 150 V, the average roughness of the coatings increases from 22.05 nm to 75.3 nm. The nanohardness of the obtained AlMgB_14_-based coatings varied from 24 to 37 GPa, depending on the RF sputtering bias voltage. The AlMgB_14_-based coating sputtered at a 100 V bias voltage had the highest nanohardness of 37 GPa along with a COF of 0.12 and a wear rate of 7.5 × 10^−5^ mm^3^/N·m. The hardness and tribological characteristics of sputtered AlMgB_14_-based coatings are in good agreement with the hardness and tribological characteristics of AlMgB_14_-based materials reported in other studies. According to the analysis of the literature, an additional increase in the wear resistance of AlMgB_14_-based coatings could be achieved by introducing up to 50 wt. % TiB_2_ in the composition. This provides a perspective for further research aimed at investigating the effect of additives, such as TiB_2_, on the structure and properties of AlMgB_14_-based coatings. In addition, further research could also include the study of AlMgB_14_-based coatings under near-operational conditions—for example, when sputtered on the surface of cutting tools or on friction pairs.

## Figures and Tables

**Table 1:** Coatings sputtering modes.

Parameter	Cleaning and Heating	Sputtering
Plasma generator current, A	15	50
Ar pressure, Pa	0.5	0.5
Bias voltage, V	990	30/50/100/150
RF-generator power, W	-	700
Time, min	15	180

**Table 2:** Comparison of the properties of the obtained AlMgB_14_-based coatings with the properties of similar materials reported in the literature.

Composition	Method of Obtaining	Conditions of the Tribological Properties Determination	Hardness, GPa	COF	Wear Rate, mm^3^/N·m	References
AlMgB_14_	RF plasma sputtering	Ball material: 100Cr6 Environment: dry Load: 1 N Speed: 25 mm/s	37 ± 2	0.12	7.5 × 10^−5^	This work
AlMgB_14_-30 wt. % Si	Spark plasma sintering	Ball material: 316 L Environment: dry Load: 60 N Speed: 60 mm/s	27.45 ± 2.15	0.19–0.28	8 × 10^−6^	[25]
AlMgB_14_-30 wt. % Si	Spark plasma sintering	Ball material: Si_3_N_4_ Environment: dry Load: 60 N Speed: 60 mm/s	27.45 ± 2.15	0.35–0.46	3.8–9 × 10^−5^	[25]
AlMgB_14_-50 wt. % TiB_2_	Magnetron sputtering	Ball material: 52,100 Steel Environment: hydraulic oil Load: 10 N Speed: 500 mm/s	17.8	0.08	-	[8]
AlMgB_14_	Pulsed laser deposition	Ball material: 52,100 Steel Environment: dry Load: 1 N Speed: 20 mm/s	-	0.2	-	[28]
AlMgB_14_-50 wt. % TiB_2_	Physical vapor deposition	Ball material: 52,100 Steel Environment: dry Load: 10 N Speed: 500 mm/s	-	0.6	6.5 × 10^−7^	[28]
Al-Mg-B powder stoichiometric composition of elements	Magnetron sputtering	Ball material: Si_3_N_4_ Environment: dry Load: 3 N Speed: 1 mm/s	30	0.2–0.4	-	[29]
Al-Mg-B-Ti-B powder stoichiometric composition of elements	Magnetron sputtering	Ball material: 100Cr6 Environment: water-glycol Load: 1–2 N Speed: 300 mm/s	-	0.02–0.05	-	[27]

## Data Availability

The data presented in this study are available in the article.

## References

[1] Cook B.A., Harringa J.L., Lewis T.L., Russell A.M. (2000). A New Class of Ultra-Hard Materials Based on AlMgB_14_. Scr. Mater..

[2] Gotman I., Gutmanas E.Y. (2014). Titanium Nitride-Based Coatings on Implantable Medical Devices. Adv. Biomater. Devices Med..

[3] Ruggiero P.F. Tungsten Carbide Coatings Replace Chromium. http://www.wococarbide.com/Uploads/2017-07-31/597edb1697a16.pdf.

[4] Cremer R., Reichert K., Neuschütz D., Erkens G., Leyendecker T. (2003). Sputter Deposition of Crystalline Alumina Coatings. Surf. Coat. Technol..

[5] Mikula M., Grančič B., Buršíková V., Csuba A., Držík M., Kavecký Š., Plecenik A., Kúš P. (2007). Mechanical Properties of Superhard TiB2 Coatings Prepared by DC Magnetron Sputtering. Vacuum.

[6] Sumant A.V., Krauss A.R., Gruen D.M., Auciello O., Erdemir A., Williams M., Artiles A.F., Adams W. (2005). Ultrananocrystalline Diamond Film as a Wear-Resistant and Protective Coating for Mechanical Seal Applications. Tribol. Trans..

[7] Cherukuri R., Womack M., Molian P., Russell A., Tian Y. (2002). Pulsed Laser Deposition of AlMgB_14_ on Carbide Inserts for Metal Cutting. Surf. Coat. Technol..

[8] Cook B.A., Harringa J.L., Anderegg J., Russell A.M., Qu J., Blau P.J., Higdon C., Elmoursi A.A. (2010). Analysis of Wear Mechanisms in Low-Friction AlMgB_14_–TiB_2_ Coatings. Surf. Coat. Technol..

[9] Higdon C.B., Cook B., Harringa J., Russell A., Goldsmith J., Qu J., Blau P. (2011). Friction and Wear Mechanisms in AlMgB_14_-TiB_2_ Nanocoatings. Wear.

[10] Nikitin P.Y., Zhukov I.A., Matveev A.E., Sokolov S.D., Boldin M.S., Vorozhtsov A.B. (2020). AlMgB_14_–TiB2 Composite Materials Obtained by Self-Propagating High-Temperature Synthesis and Spark Plasma Sintering. Ceram. Int..

[11] Zhukov I.A., Nikitin P.Y., Vorozhtsov A.B., Perevislov S.N., Sokolov S.D., Ziatdinov M.H. (2020). The Use of Intermetallic AlxMgy Powder to Obtain AlMgB_14_-Based Materials. Mater. Today Commun..

[12] Kevorkijan V., Škapin S.D., Jelen M., Krnel K., Meden A. (2007). Cost-Effective Synthesis of AlMgB_14_–XTiB2. J. Eur. Ceram. Soc..

[13] Ivashchenko V.I., Scrynskyy P.L., Dub S.N., Butenko O.O., Kozak A.O., Sinelnichenko O.K. (2016). Structural and Mechanical Properties of Al―Mg―B Films: Experimental Study and First-Principles Calculations. Thin Solid Films.

[14] Grishin A.M., Khartsev S.I., Böhlmark J., Ahlgren M. (2015). Ultra-Hard AlMgB_14_ Coatings Fabricated by RF Magnetron Sputtering from a Stoichiometric Target. JETP Lett..

[15] Noroozi M., Petruhins A., Greczynski G., Rosen J., Eklund P. (2020). Structural and Mechanical Properties of Amorphous AlMgB_14_ Thin Films Deposited by DC Magnetron Sputtering on Si, Al_2_O_3_ and MgO Substrates. Appl. Phys. A Mater. Sci. Process..

[16] Yan C., Qian J.C., Ng T.W., Zhou Z.F., Li K.Y., Zhang W.J., Bello I., Martinu L., Klemberg-Sapieha J.E. (2013). Sputter Deposition of Hard Quaternary Al–Mg–B–Ti Nanocomposite Films. Surf. Coat. Technol..

[17] Nikitin P.Y., Matveev A.E., Zhukov I.A. (2021). Energy-Effective AlMgB_14_ Production by Self-Propagating High-Temperature Synthesis (SHS) Using the Chemical Furnace as a Source of Heat Energy. Ceram. Int..

[18] Shugurov V.V., Akhmadeev Y.H., Zhukov I.A., Yu Nikitin P. (2021). Deposition of AlMgB_14_ Films by Sputtering in a Non-Self-Sustained High-Frequency Discharge. J. Phys. Conf. Ser..

[19] Oliver W.C., Pharr G.M. (1992). An Improved Technique for Determining Hardness and Elastic Modulus Using Load and Displacement Sensing Indentation Experiments. J. Mater. Res..

[20] Tkachev D., Nikitin P., Zhukov I., Vorozhtsov A., Marchenko E., Verkhoshanskiy Y., Belchikov I. (2023). Structure and Flexural Strength of the Hot-Pressed AlMgB_14_ Ceramic. Phys. Scr..

[21] Grishin A.M. (2020). Hardness, Young’s Modulus and Elastic Recovery in Magnetron Sputtered Amorphous AlMgB_14_ Films. Crystals.

[22] Hui Z., Li Z., Ju P., Yingjian N., Ouyang J., Tian Y. (2019). Comparative Studies of the Tribological Behaviors and Tribo-Chemical Mechanisms for AlMgB_14_-TiB_2_ Coatings and B4C Coatings Lubricated with Molybdenum Dialkyl-Dithiocarbamate. Tribol. Int..

[23] Werheit H., Filipov V., Kuhlmann U., Schwarz U., Armbrüster M., Leithe-Jasper A., Tanaka T., Higashi I., Lundström T., Gurin V.N. (2010). Raman Effect in Icosahedral Boron-Rich Solids. Sci. Technol. Adv. Mater..

[24] Tian Y., Bastawros A.F., Lo C.C.H., Constant A.P., Russell A.M., Cook B.A. (2003). Superhard Self-Lubricating AlMgB_14_ Films for Microelectromechanical Devices. Appl. Phys. Lett..

[25] Chen J., Cheng J., Li F., Zhu S., Li W., Yang J., Liu W. (2017). Tribological Study on a Novel Wear-Resistant AlMgB_14_-Si Composite. Ceram. Int..

[26] Nikitin P., Zhukov I., Tkachev D., Abzaev Y., Marchenko E., Vorozhtsov A. (2022). Experimental and Theoretical Study of Ultra-Hard AlMgB_14_-TiB_2_ Composites: Structure, Hardness and Self-Lubricity. Materials.

[27] Lu X., Yao K., Ouyang J., Tian Y. (2015). Tribological Characteristics and Tribo-Chemical Mechanisms of Al–Mg–Ti–B Coatings under Water–Glycol Lubrication. Wear.

[28] Qu J., Blau P.J., Zhu D., Cook B.A., Elmoursi A.A. Tribological Characteristics of AlMgB_14_ and Nanocomposite AlMgB_14_-TiB_2_ Superhard Coatings. Proceedings of the STLE/ASME 2008 International Joint Tribology Conference.

[29] Qu W., Wu A., Wu Z., Bai Y., Jiang X. (2012). Influence of Boron Contents on Properties of AlMgB Films Prepared by RF Magnetron Sputtering. Rare Metals.

